# Conditional cash transfers for primary education: Which children are left out?

**DOI:** 10.1016/j.worlddev.2017.12.021

**Published:** 2018-05

**Authors:** Jonathan Bauchet, Eduardo A. Undurraga, Victoria Reyes-García, Jere R. Behrman, Ricardo A. Godoy

**Affiliations:** a Department of Consumer Science, Purdue University, 812 W. State Street, West Lafayette, IN 47906, USA; b School of Government, Pontificia Universidad Católica de Chile, Av. Vicuña Mackenna 4860, CP 7820436 Santiago, RM, Chile; c Institució Catalana de Recerca i Estudis Avançats (ICREA), Passeig Lluis Companys 23, 08010 Barcelona, Spain; d Institut de Ciència i Tecnologia Ambientals, Universitat Autònoma de Barcelona, 08193 Bellaterra, Spain; e Departments of Economics and Sociology and Population Studies Center, University of Pennsylvania, 229 McNeil Building, 3718 Locust Walk, Philadelphia, PA 19104, USA; f Heller School for Social Policy and Management, Brandeis University, 415 South Street, Waltham, MA 02453, USA

**Keywords:** Bolivia, *Bono Juancito Pinto*, Gender disparity, Human capital, Indigenous people

## Abstract

Conditional cash transfer (CCT) programs to increase primary-school enrollment and attendance among low-income households have been shown to benefit children and households, but to date little is known about who joins such programs. We test three hypotheses about predictors of CCT program participation in indigenous societies in Bolivia, focusing on attributes of the household (ethnicity), parents (modern human capital), and children (age, sex). We model whether children receive a transfer from Bolivia’s CCT program (*Bono Juancito Pinto*), using data from 811 school-age children and nine ethnic groups. Children from the group least exposed to Westerners (Tsimane’) are 18–22 percentage points less likely to participate in the program than children from other lowland ethnic groups. Parental modern human capital and child sex do not predict participation. We discuss possible mechanisms underlying the findings and conclude that the Tsimane’s current lower returns to schooling are the most likely explanation.

## Highlights

• We investigate predictors of child participation in Bolivia’s CCT program.
• Children less exposed to Westerners have lower probabilities of receiving transfers.
• Participation rates are highest around age 11 y.
• Parents’ modern human capital is not associated with participation.
• Participation rates are similar for boys and girls.

## 1 Introduction

Since their first implementation in Brazil and Mexico in 1997, conditional cash transfer (CCT) programs to increase primary school enrollment and attendance among children from low-income households have spread widely in Latin America (Behrman et al., 2011, Handa and Davis, 2006, Lomeli, 2008, Nagels, 2016) and beyond (Behrman, 2010, Fiszbein and Schady, 2009, Saavedra and García, 2013). In most CCT programs, parents or children receive cash payments if the child attends school for a minimum of about 80–85% of school days (Fiszbein & Schady, 2009, p. 86). Evaluations of CCT programs show that they have met their goals of increasing school enrollment and attendance (Baird et al., 2014, Behrman et al., 2005, Benedetti et al., 2016, Fiszbein and Schady, 2009, Ganimian and Murnane, 2016, Schultz, 2004, Todd and Wolpin, 2006), but also show that their effects on academic and cognitive skills remain unclear (Baird et al., 2011, Ganimian and Murnane, 2016, McEwan, 2015).

Despite the voluminous literature on the impacts of CCT programs in Latin America, little is known about their impacts among indigenous peoples, who are among the poorest in the continent (Hall & Patrinos, 2012; Van de gaer, Vandenbossche, & Figueroa, 2013). The few published impact evaluations of CCT programs among indigenous peoples come from Mexico’s CCT programs, and show that the programs increased primary-school enrollment and attendance (López-Calva & Patrinos, 2015) and some cognitive skills (Figueroa, 2014), but failed to improve school drop-out (Álvarez, Devoto, & Winters, 2008).

Even less is known about predictors of indigenous peoples’ participation in CCT programs. Collateral evidence from evaluations of such programs among disadvantaged groups suggests that children from low-income households find it hard to access the programs owing to the costs of compliance and transport to school, parental misperceptions about the program, and low parental modern human capital (i.e., literacy) (Cecchini and Madariaga, 2011, de Janvry and Sadoulet, 2006, Lomeli, 2008, McGuire, 2013).

Indigenous peoples in Latin America on average underperform in comparison with their non-indigenous peers in educational attainment (Arteaga and Glewwe, 2014, Gigler, 2015, Hall and Patrinos, 2012, Reimão and Taş, 2017, Valenzuela, 2004). In Bolivia, McEwan (2004) estimates that primary school students from all indigenous groups perform 0.3–0.5 standard deviations (SD) worse on standardized tests of mathematics and Spanish than their non-indigenous peers. Reimão and Taş (2017) used 2012 Bolivian census data to estimate the gap in school achievement between non-indigenous adults and adults from minority indigenous groups (i.e. not Quechua and Aymara, the two largest indigenous groups in Bolivia). They found that women and men from minority indigenous groups completed 3.3 and 2.9 fewer grades of schooling than their peers from non-indigenous groups. These trends hold in our sample: children between seven and 16 years of age from the most isolated groups (Tsimane’) completed 2.5 fewer years of schooling than children from less isolated groups.

Since CCT programs to encourage primary-school enrollment and attendance can produce both lasting effects on children and spillover effects within the household (Behrman and Parker, 2013, Behrman et al., 2011), and as indigenous peoples underperform in educational attainment and are among the most marginalized groups in Latin America, identifying the predictors of program participation matters when designing better ways of reaching marginalized indigenous peoples.

This article has three aims: (1) to characterize inter-ethnic and intra-ethnic variation in participation in a CCT program for primary-school enrollment and attendance in several native Amazonian societies of Bolivia, (2) to test hypotheses about household, parental, and child characteristics associated with participation in such a program, and (3) to explore mechanisms that explain differences in participation. Like many other Latin American countries, Bolivia started a CCT program to encourage primary-school attendance. Known as *Bono Juancito Pinto*, the program since its inception in 2006 has paid annually US$28 per child attending grades 1–5, provided the child attends a public school and misses no more than 20% of school days in the academic year. Children (or their households) are eligible to receive transfers irrespective of household income, with payments made at the end of the school year for qualifying children (McGuire, 2013). Since its beginning, the program has expanded to cover up to the sixth (2007), eighth (2008), ninth (2012), and twelfth (2014) grades (McGuire, 2013).

Unlike some other educational CCT programs, Bolivia’s CCT program has not been evaluated with a randomized controlled trial. National statistics suggest that the program’s impacts are mixed. On the one hand, as the number of beneficiaries of the program increased from 1.1 million in 2006 to 2.2 million in 2016 (Ministerio de Economía y Finanzas Públicas, 2016), national enrollment rates have decreased. According to the latest data from the Institute for Statistics of UNESCO, the net primary-school enrollment rate in Bolivia declined every year from 96% when the program started in 2006 to 89% in 2015, the last year for which information was available (UNESCO, 2016). Aguilar Pacajes (2014) shows that the decline in primary-school enrollment was sharper in rural areas and in public schools than in cities or than in private schools (the CCT program does not cover children enrolled in private schools). On the other hand, rates of primary-school graduation have been increasing. The share of children completing primary school has increased from 80% in 2006 to 97% in 2014, and the proportion of children enrolled in the last year of primary school who transition to secondary school has increased from 93% in 2006 to 97% in 2014 (UNESCO, 2016). The share of children in primary schools who continue their education has also increased in rural areas, with girls outperforming boys, although rural areas continue to lag behind urban areas (Aguilar Pacajes, 2014). To date, little is known about how Bolivia’s CCT program for school enrollment and attendance works among indigenous peoples.

We analyze cross-sectional data on 811 children from multiple ethnic groups in a rural area of lowland Bolivia and find that children from the more-isolated, less-acculturated group (Tsimane’) are less likely to receive a payment from the CCT program, and that child age has a non-linear relation with the likelihood of receiving the CCT payment. Children of about 11 years of age are most likely to take part in the program. On the other hand, parental characteristics, child sex, and school characteristics are not associated with the likelihood that a child receives a payment. We examine several possible explanations for the findings and conclude that the main mechanism at work is the lower returns to modern schooling for Tsimane’ households.

## 2 Hypotheses

The analysis centers on testing three hypotheses about predictors of CCT program participation among indigenous people in the Bolivian Amazon. The hypotheses focus on attributes of the household (ethnicity), parents (modern human capital), and children (age, sex) in grades ≤ 8.

H1 **Ethnic attributes**

*After controlling for household, child, and school attributes, children from less-acculturated ethnic groups are less likely to participate in the CCT program for primary-school attendance than children from more-acculturated ethnic groups.*

We use the term acculturation as a synonym for degree of incorporation into Western national society. Bolivian native Amazonian societies vary in their mode of incorporation into national society, from groups that historically avoided contact with outsiders to groups that engaged with Westerners and Catholic missions as early as the sixteenth century (Godoy et al. 2015; 2005). Our sample (described later) captures that range; it contains native Amazonian societies with centuries of close contact with Jesuit missions and ranchers (Moxeños), to cosmopolitan indigenous people originally from the highlands, to groups like the Tsimane’ who have kept arm’s-length relations with Westerners. 83% of the 24 villages studied included individuals of more than one ethnic group. Since our sample is mostly composed of ethnically-mixed villages, we can test whether people from different ethnic groups sharing the same village school and having the same access to market towns vary in their propensity to take part in the educational CCT program.

H2 **Parental modern human capital**

*Children from households in which parents have higher levels of modern human capital are more likely to enroll in the CCT program.*

We equate modern human capital with school attainment and with Spanish-speaking fluency. We expect to see a positive correlation between *(i)* parental modern human capital and *(ii)* the probability that children receive CCT payments. A national identification card makes it easier for children to receive the transfer, since without it children would need two persons from the community vouching for their identity to receive the transfer (McGuire, 2013). Since obtaining a national identification card entails transaction costs (e.g., trips to towns) and the ability to deal in Spanish with governmental officials, the requirement may act as a filter for selecting children from households with more-schooled, Spanish-speaking parents. The hypothesis also builds on findings from other studies in Latin America that show that some eligible households lack the resources to obtain the documentation to access educational CCT programs (Feitosa de Britto, 2004) and from studies in Bolivia showing that adult Spanish-speaking fluency among indigenous peoples is associated with better market returns (Chiswick et al., 2000, Godoy et al., 2007, Saidi et al., 2016).

H3 **Child attributes**

*(a) There is no girl-boy disparity in participation in the CCT program. (b) Older children are more likely to enroll in the CCT program than younger siblings.*

These hypotheses build on previous work among the Tsimane’. (a) Previous research among the Tsimane’ suggests no girl-boy difference in school grades completed, academic skills, or in anthropometric indicators of nutritional status (Godoy et al., 2006). (b) In a previous study we found that Tsimane’ school-aged girls or boys (5 years < age < 16 years) with an older sibling, particularly an older brother, are less likely to enroll in school and complete fewer grades of schooling (Zeng et al., 2012). We build on these results for H3, aware that findings from the Tsimane’ might lack external validity among neighboring ethnic groups.

## 3 Sample and study participants

**Fig. 1:**
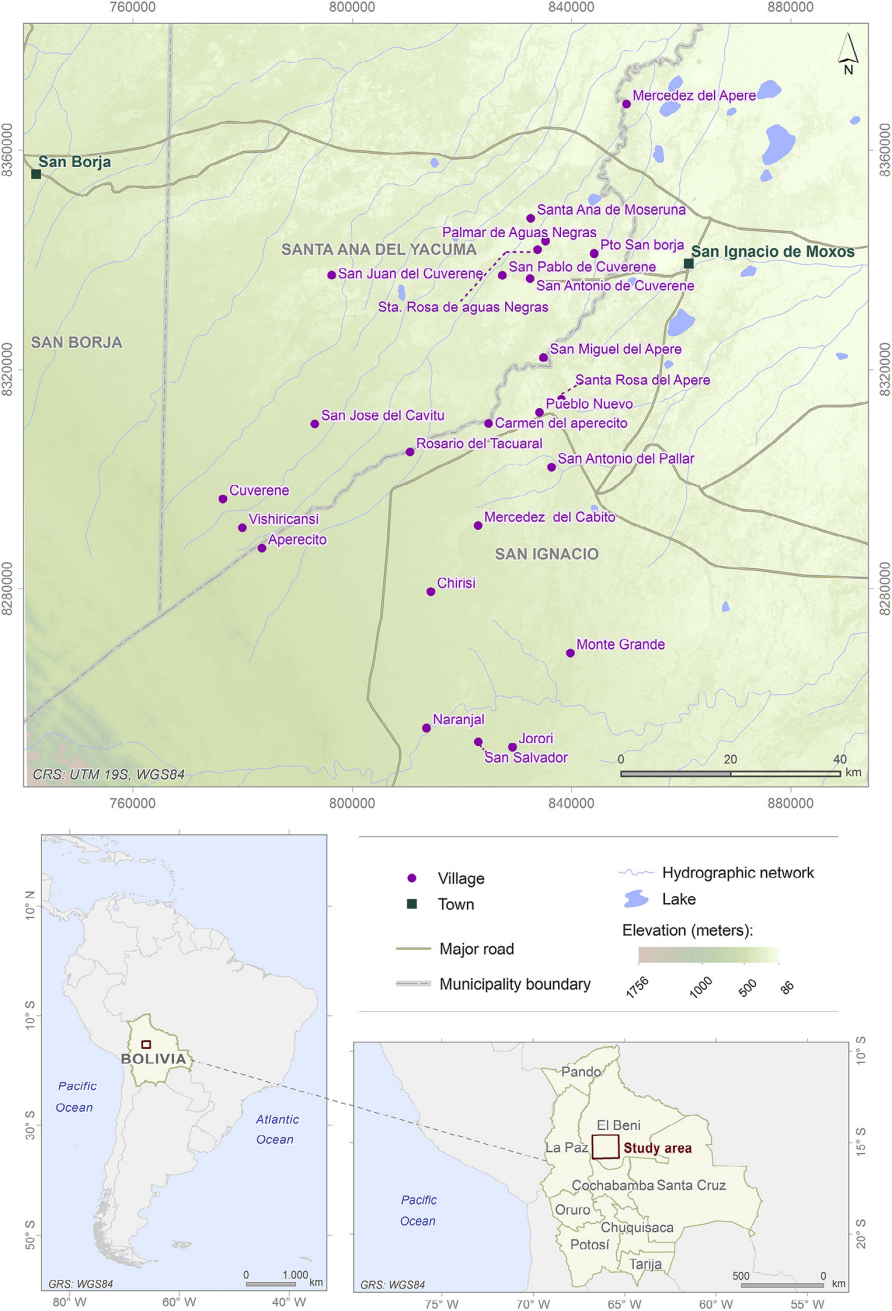
Map of the study area (Territorio Indígena Multiétnico, Dept. of Beni, Bolivia). Source: elaborated with cartographic data from Digital Chart of the World, GDAM and GeoBolivia (ASTER GDEM) repositories. Digital Chart of the World: http://worldmap.harvard.edu/data/geonode:Digital_Chart_of_the_World. GDAM: http://www.gadm.org/. GeoBolivia: http://geo.gob.bo/#map.

The study took place in the Territorio Indígena Multiétnico (TIM), department of Beni (Fig. 1). The area has 25 villages, but one village did not have a school in 2012, so the educational CCT program was not implemented in that village. We dropped this village from the sample. Thus, we use 24 villages for the analyses. Table 1 contains a summary of the number of villages, households, individuals, and children included in the analysis.

**Table 1:** Sample of villages, households, and people used in the analysis.

Villages	24

Households	620

Individuals (all ages)	2922

Children	811

Children in the sample are 7 years ≤ age≤16 years, attended primary school (grade ≤ 8), and have non-missing data on CCT participation.

A team with extensive experience doing household surveys among Bolivian native Amazonians conducted the survey (Leonard et al., 2015). The survey focused on household socioeconomic conditions and was directed at household heads, but included a demographic module with questions about the age, sex, and school-grade attainment of all children in the household, including whether each child had received a payment from the CCT program the previous year.

The main form of subsistence in the area centers on slash-and-burn cultivation, supplemented with fishing, hunting, plant collection, and wage labor in cattle ranches and logging camps. Our survey suggests that mean household monetary income per person from the sale of goods and wage labor is US$1.04/day (median = $0.17, SD = 2.1), but 39% of households in the sample earn no monetary income (1 US$ = 7 *bolivianos*). Daily monetary income per person varies between ethnic groups, from a low of US$0.50 among the Tsimane’, to US$1.14 for Moxeños, to US$1.71 among the other indigenous groups and non-indigenous lowlanders. 78% of the children in our sample lived in extreme poverty (<US$1.25/person/day) and 90% lived in poverty (<US$2.50/person/day). These figures compare unfavorably with national averages for 2011. According to Vargas and Garriga (2015), in 2011 7% of people in Bolivia lived in extreme poverty and 16% lived in poverty (see also UDAPE, 2015, p. 25).

During the study, six native Amazonian lowland groups inhabited the area (Trinitario, Ignaciano, Moxeño, Tsimane’, Yuracaré, Movima), in addition to indigenous highlanders (colla) and non-indigenous lowlanders (camba) (Stearman, 1985). For the analysis, we lump Trinitario, Ignaciano, and Moxeño under the rubric Moxeño since they often use this term to refer to themselves as a collective. The three groups have had long exposure to Jesuit missions and Westerners (Block, 1994), and in the late 19th century and early 20th century they took part in the rubber boom as indentured workers (Vallvé, 2010, van Valen, 2013) and in a millenarian religious movement (Lehm Ardaya, 1999, Riester, 1976). Fluent in Spanish, literate, and acculturated into the livestock culture of the Jesuit missions, they soon became useful ranch-hands for Spanish cattle owners. Today, the Trinitarios, Ignacianos, and Moxeños are among the most acculturated lowland groups.

We lump the Yuracaré and Movima and refer to them collectively as a Lowland group. They too have had long exposure to outsiders (the Yuracaré less so than the Movima), but neither group has had as much exposure to outsiders as the Moxeño. Most Yuracaré and Movima are bilingual in their tongue and in Spanish and are well-integrated into the market economy, often as ranch hands (Thomas, 2012).

People from the lowlands use the term colla (sometimes pejoratively) to refer to highlanders of Aymara or Quechua stock (Stearman, 1985). Highland-lowland economic and social relations go back to pre-Hispanic days (Godoy, 2015), but the large influx of highlanders into the territory of native Amazonians started during the 1970s as the government set up colonization projects in the lowlands to ease population pressure in the highlands (Jones, 1995). At present, collas in the lowlands play a prominent role in transport and trade.

Last, the Tsimane’ have been extensively described in the anthropological literature (e.g., Huanca, 2008, Reyes-García and Huanca, 2015, Ringhofer, 2010) and form the group historically least exposed to outsiders, although levels of exposure have increased in recent decades (Reyes-García et al., 2014). In a worldwide comparative study of 15 small-scale rural societies, the Tsimane’ ranked next to last in market interactions, with only 7% of total household calories bought in the market (Henrich et al., 2010).

## 4 Bolivia’s conditional cash transfer program for primary education (*Bono Juancito Pinto*): A view from the lowlands

McGuire (2013) discusses the origins and development of Bolivia’s educational CCT program, *Bono Juancito Pinto,* and elsewhere we discuss how the educational system works in one of the groups considered (Tsimane’) in a neighboring area (Godoy et al., 2007, Reyes-Garcia et al., 2010, Undurraga et al., 2013), so in this section we focus on how the CCT program works in the study area.

Bolivia’s educational system is officially divided into an obligatory first stage and an optional second stage. The first stage covers primary education and includes grades 1–8, broken down into three parts: basic (grades 1–3), intermediate (grades 4–6), and applied (grades 7–8). After primary education, children enter two two-year cycles of high school education covering grades 9–10 and 11–12. In rural areas, grades 13–15 center on technical university careers.

The 24 villages we analyze are in relatively inaccessible areas, far from roads or towns (Table 2). Only seven villages were accessible year-round by motorized vehicles in 2012. The typical school has two teachers, at least one latrine, and a school committee that had met at least once in the year before the survey. Most schools have a breakfast program (92%) and multi-grade classrooms (96%). Since villages are typically small (mean = 28 households/village, SD = 19), teachers know first-hand their students and their families. In most schools surveyed classes are taught only in the morning. In larger villages and in grades 6–8, schools run in two shifts, from 8am until noon, and from 2 pm until 6 pm; in those villages children attend only one of the two shifts. Thus, all children have available time to help with farm and household chores when not in school.

**Table 2:** Attributes of villages and schools as reported by village authorities (n = 24).

Attribute	Mean	SD^a^	Median
*A. Villages*			
Number of households in a village	28.0	18.6	20
Village accessible by motorized vehicles all year	29%		
In dry season, hours walking from village to nearest road	9.1	9.3	5
In dry season, hours walking from village to nearest town	14.9	9.8	10

*B. Schools*^b^			
Share of households in village with school-aged children	59%		
Number of registered school-age children in school	51	65	29
Share of households with school-age children that do not send their children to school	13%		
Age in years of village school	21	16	22.5
Maximum grade taught in village school	6.9	3.2	6
Number of teachers in school	4.2	4.9	2
Share of schools with multi-grade classrooms	96%		
Number of children who study outside the village	5.9	8.3	2
Share of schools with a breakfast program	92%		
Number of school latrines per school	1.3	1	1
Share of schools where school committee met this year	92%		
Share of villages with an adult educational program	17%		

a SD = standard deviation.

b These variables were used in the principal component analysis to create the index of school quality.

CCT payments are made at the end of each school year (December) by the Bolivian military, on behalf of the Ministry of Education. Information about the exact dates is announced in schools and via local radio stations. Payment days are festive occasions, with parents and children arranging their schedule to ensure they are present when the army arrives. The school principal shows the head of the military unit the list of children who have complied with the obligation of attending at least 80% of school days. During the school year, the village teacher can decide if an absence is legitimate (e.g., from illness), so children who miss more than 20% of school days may still receive a payment. The army pays children, unless parents step forward on behalf of their children. Even when parents receive payments, they often hand the payment to their children because they feel that children “[have] earned it” (Zycherman, 2013, pp. 79–80). In our data, 60% of households reported that the use of the payments was decided by “other (for example, child)” – the questionnaire explicitly suggested children as possible other deciders. Children are less likely to be deciders in Tsimane’ households than in households of other ethnicities (44% versus 60%-80%). If children or parents are absent, the school principal receives the money on behalf of the student, but signs a form pledging to hand over the money to the student or parents when they return to the village.

## 5 Model and data

### 5.1 Model

To test our hypotheses, we relate receiving a payment from *Bono Juancito Pinto* to several characteristics of the households, the parents, and the children, controlling for school quality. Our sample includes 811 children meeting the sample inclusion criteria described below and for whom we have information on whether they received payment from the CCT program the year before the survey. Our primary regression equation is:

(1) $${\text{CCT}}_{\text{ihv}}=\alpha +\beta *{\text{E}}_{\text{hv}}+\theta *{\text{P}}_{\text{hv}}+\gamma *{\text{C}}_{\text{ihv}}+\delta *{\text{S}}_{\text{v}}+\varepsilon_{\text{ihv}}\text{.}$$

In Eq. (1) *i, h, and v* index children, households, and villages, CCT is a binary variable equal to one if child *i* received the CCT the previous school year, E is a vector of binary indicators of the household’s ethnicity, P is a vector of parental modern human capital indicators, C is a vector of children’s characteristics, S is an index of school characteristics, and ε indicates the error term. In one regression, we also include village fixed effects to show that our conclusions persist after controlling for village characteristics beyond those directly pertaining to school quality. Table 3 contains definition of these variables. We estimate the parameters of Eq. (1) using probit regressions, with standard errors clustered by household. In the main tables we show average marginal effects, but in the tables of the Appendix we show probit coefficients for models that include interaction terms.

**Table 3:** Definition of variables used in regression analysis.

Variable	Definition
*A. Outcome as reported by parents*	
CCT	Child received conditional cash transfer (CCT) payment as part of the *Bono Juancito Pinto* program (1); 0 otherwise. Sample includes only school-aged children (7 years ≤ age≤16 years) and primary school grades (grade ≤ 8)

*B. Explanatory variables: Child attributes as reported by parents*	
Age	Child’s age in years at the time of the survey
Girl	Child’s sex: 1 = girl; 0 = boy
Schooling	Maximum school grade completed [Not included in regressions]

*C. Explanatory variables: Self-reported parental attributes*	
Ethnicity	Dummy variables for the ethnic self-identification of parents: *Moxeño: 1* = both parents are Trinitario, Ignaciano, or Moxeño; 0 otherwise *Tsimane’: 1* = both parents are Tsimane’; 0 otherwise *Lowland: 1* = both parents are Yuracaré or Movima; 0 otherwise [excluded category in regressions] *Other: 1* = both parents are from the highlands (colla), camba/napo, or other; 0 otherwise [excluded category in regressions] *Mixed: 1* = Mother and father do not share the same ethnicity; 0 otherwise [excluded category in regressions]
Schooling	Parental maximum school grade completed. Mother’s and father’s schooling differs in 179 households; the mother had more education in 89 households, and the father in 189 households
Spanish	Parental Spanish fluency: 1 = either parent has moderate or complete fluency in spoken Spanish as judged by surveyors; 0 = both parents are monolingual in an Amazonian language

*D. Explanatory variable: School characteristics as reported by village authorities*	
School quality	Principal component factor analysis of the 12 attributes of village schools listed in Table 2, Section B. Cronbach’s alpha = 0.83; Eigenvalue of first factor = 4.74

### 5.2 Sample

For the main analysis we restrict the sample in two ways. First, we analyze data only from children 7 years ≤ age ≤ 16 years. Children typically enter school at age six; our survey asks about receiving the CCT payment the previous year, so we analyze children who were seven years and older in 2012. Sixteen is the age at which Tsimane’ children typically marry and form new households; this age might not apply to children from other ethnic groups, but applying it uniformly standardizes our analysis. Later we perform sensitivity analysis to see if results change when we raise the top age. Second, we drop children whose education level is higher than grade nine; we fielded the survey in 2012 and asked about receiving a transfer from the CCT program the year before, so we include all children who were in grades covered by the program that year. We assume that no child in our sample repeated the ninth grade, which we cannot verify with our data. Forty children meet our sample inclusion criteria but had missing information on whether they received the transfer so they are not included in the analysis. These children are from households that speak less Spanish, are older, and are more likely to be girls than the 811 children in our final sample.

### 5.3 Summary statistics

Table 4 provides summary statistics on the full sample, disaggregated by participation in the CCT program. The average age of children in the sample is 11 years. In the regressions we include both age and age squared because the relation between CCT participation and age is non-linear: participation increases for children up to age 13 and decreases for older children (Appendix Fig. 1; the predicted participation rate from the regressions increases up to age 11). The sample is almost evenly split between girls (47%) and boys (53%). On average, children completed 3.9 grades of school, but children who did not participate completed fewer grades (2.9) than children who did participate (4.1; p < .001). The gap between participants and non-participants is wider for younger children than older children. This age pattern implies that, unlike *PROGRESA/Oportunidades* in Mexico where little impact on enrollment was found for younger children because enrollment rates were already high (Behrman et al., 2005, Parker and Todd, 2017, Schultz, 2004), the Bolivian educational CCT program can be expected to have large impact on enrollment, attendance, and grade progression.

**Table 4:** Summary statistics by CCT program participation.

	Total (n = 811)		Received CCT (n = 695)		Did not receive CCT (n = 116)		p-value from t tests
	Mean	SD	Mean	SD	Mean	SD	
*A. Child*							
Age	11	2.6	11	2.4	11	3.3	0.138
Girl (%)	47		48		41		0.223
Schooling (grades)	3.9	2.4	4.1	2.2	2.9	2.7	<0.001

*B. Mother*							
Ethnicity (%)							
*Moxeño*	77		81		56		<0.001
*Tsimane’*	14		10		35		<0.001
*Lowland*	5		6		1		0.027
*Other*	4		4		8		0.042
Schooling (grades)	3.8	3.4	4.0	3.3	2.8	3.5	0.001
Speaks Spanish (%)	81		85		58		<0.001

*C. Father*							
Ethnicity (%)							
*Moxeño*	74		77		54		<0.001
*Tsimane*’	13		10		35		<0.001
*Lowland*	8		8		6		0.504
*Other*	5		5		5		0.935
Schooling (grades)	4.6	3.8	4.8	3.7	3.6	3.8	0.006
Speaks Spanish (%)	82		85		63		<0.001

*Village school quality*							
Children attending school in top 50% of school quality (%)	77	42	81	39	51	50	<0.001

Table shows means and standard deviations (SD) by whether child in primary school (grades ≤ 8; 7 years ≤ age ≤ 16 years) received the CCT educational payment from *Bono Juancito Pinto* in 2011. For definitions of variables see Table 3. Numbers of observations are lower than 811 for mothers’ and fathers’ characteristics due to missing values. P values are from t-tests of the difference in means between children receiving and not receiving the CCT. The village-level village school quality index has a mean of 0 and a standard deviation of 1 by construction; the index values range from −1.21 to 3.56.

Parental characteristics include parents’ ethnicity, maximum school grade completed, and Spanish-speaking ability. 77% of mothers and 74% of fathers are Moxeño, about 13% of both mothers and fathers are Tsimane’, and the remainder are lowlanders or people from other ethnic groups described above. In the regression analyses the binary variables indicating households’ ethnicity are equal to one if both parents are of the same ethnicity and zero otherwise; 86% of couples are of the same ethnicity. Mixed-ethnicity couples are indicated by a separate binary variable. 70% of all children are from Moxeño households, 13% are from Tsimane’ households, 13% are from mixed-ethnicity households, and the remainder have parents from other ethnic groups. Parents have on average only completed about half of the primary school grades. Mothers completed 3.8 grades of schooling, and fathers completed 4.6 grades. About 80% of both parents speak Spanish, although parents of children receiving a transfer were statistically significantly more likely to speak Spanish.

In the regressions we control for school quality, measured by an index that we created. The index of school quality is a Z-score from the principal component factor analysis of 12 attributes of village schools, described in Table 3. Table 4 shows that children who do not receive the payment live in villages with schools of lower quality than children who receive the payment. Only 51% of those who do not receive a payment live in a school ranked in the top half of quality, according to our index, against 81% of those who receive a payment.

### 5.4 Comparison of summary statistics by ethnic group

In Table 5 we compare characteristics of children, parents, and villages by ethnic group. Several messages arise from the table. First, rates of participation in the CCT program are lower for Tsimane’ children than for children of all other ethnic groups. 62% of Tsimane’ children received a payment from the CCT program, but 83% to 94% of children of other ethnic group received the payment.

**Table 5:** Summary statistics, by parents’ ethnicity.

Couple’s ethnicity:	Tsimane’		Moxeño		Lowland		Other		Mixed	
	N	Mean	N	Mean	N	Mean	N	Mean	N	Mean
*A. Child*										
Received CCT (%)	90	62	502	90	17	94	12	83	89	88
Age	90	10	502	11	17	12	12	11	89	11
Girl (%)	90	47	502	48	17	41	12	58	89	52
Schooling (grades)	90	1.7	502	4.3	17	4.9	12	4.7	89	3.7

*B. Mother*										
Schooling (grades)	89	0.4	491	4.4	15	3.3	12	2.8	89	3.8
Speaks Spanish (%)	89	3	491	93	15	100	12	100	89	89

*C. Father*										
Schooling (grades)	87	0.9	451	5.2	17	4.3	12	6.2	80	4.9
Speaks Spanish (%)	87	10	451	93	17	100	12	100	80	93

*D. Villages*										
Number of households	90	18	502	44	17	36	12	54	89	39
Accessible all year (%)	90	3	502	43	17	65	12	58	89	52
Hours walking from village to nearest town	89	21	341	12	17	8	10	11	80	10
Children attending school in top 50% of school quality (%)	90	3	502	89	17	65	12	83	89	73

Table shows variables used in main regression analysis, by parents’ ethnicity. For definition of variables see Table 3. Statistical tests of the differences in means across ethnic groups (ANOVA for continuous variables or chi-squared tests for categorical variables) are all significant at the 1% level. The village-level school quality index has a mean of 0 and a standard deviation of 1 by construction; the index values range from –1.21 to 3.56.

Second, both Tsimane’ children and adults have lower schooling and fluency speaking Spanish than members of other ethnic groups, or than people from mixed-ethnicity households. For example, Tsimane’ children have completed 1.7 school grades, but children of other ethnic groups have completed 3.7 to 4.9 school grades. Perhaps the most striking difference between Tsimane’ and other ethnic groups is in the Spanish-speaking ability of mothers: only 3% of Tsimane’ mothers speak some or fluent Spanish, versus 89% to 100% of mothers from other ethnic groups.

Finally, Table 5 shows that – compared with children in other groups – Tsimane’ children live in smaller, more remote villages than children of other ethnic groups. Tsimane’ children live in villages with about 18 households, located 21 hours on foot from the nearest town. Only 3% of villages are accessible year-round. Tsimane’ children also attend lower-quality schools. Our index of school quality is statistically significantly different for children of various ethnicities. Tsimane’ children are the least likely to live in a village in which the school is in the top 50% of our quality index (3%), whereas 65% to 89% of children of other ethnic groups live in villages in which the school is in the top 50% of our quality index.

### 5.5 Multicollinearity

The relation between ethnicity, schooling and Spanish fluency suggest that our regressions, which include all these variables, could suffer from multicollinearity. Indeed, we show in Appendix Table 1 that some of the variables included in our regressions are strongly correlated with each other. Multicollinearity does not appear to affect our results, however, since variance inflation factors (VIFs) for variables in our main regressions are lower than 10, used as a rule of thumb to detect problematic multicollinearity (Hair, 2010, Kutner, 2004), in nearly all our regressions (Appendix Table 2). In addition, our estimated coefficients for variables other than parental modern human capital do not change if either or both of the variables for parental modern human capital (schooling and fluency speaking Spanish) are omitted (Appendix Table 3).

VIFs for age and age squared are high, because these are naturally highly correlated, but drop to 4.14 or below if age squared is not included in the regressions. In regressions including binary variables controlling for the village in which children live (Table 6, column 5), the VIFs for the school quality index are high (about 73) because there is typically only one school per village. This result is not worrisome because regression coefficients in Table 6, column 5, are similar in magnitude, sign, and statistical significance to the other coefficients without the full set of village binary variables.

**Table 6:** Correlates of child participation in CCT program of primary-school attendance.

	(1)	(2)	(3)	(4)	(5)
Dependent variable:	1 if child received payment from CCT program; 0 otherwise				
*H1: Ethnic attributes – Household ethnicity*					
Parents are both Tsimane’	−0.200^***^	−0.216^***^	−0.177^***^	−0.216^***^	−0.175^**^
	(0.056)	(0.052)	(0.060)	(0.054)	(0.079)
Parents are both Moxeño	0.020				
	(0.035)				

*H2: Parental modern human capital*					
Max. schooling of parents	−0.001	−0.001		−0.003	−0.001
	(0.005)	(0.005)		(0.005)	(0.005)
Max. Spanish fluency of parents	−0.026	−0.026		0.001	−0.094^*^
	(0.053)	(0.052)		(0.056)	(0.053)
Mother: Schooling			−0.008		
			(0.006)		
Mother: Speaks Spanish			0.018		
			(0.094)		
Father: Schooling			0.005		
			(0.005)		
Father: Speaks Spanish			0.021		
			(0.101)		

*H3: Child attributes*					
Age (years)	0.279^***^	0.280^***^	0.280^***^	0.269^***^	0.286^***^
	(0.035)	(0.035)	(0.038)	(0.035)	(0.035)
Age squared	−0.012^***^	−0.012^***^	−0.013^***^	−0.012^***^	−0.013^***^
	(0.002)	(0.002)	(0.002)	(0.002)	(0.002)
Girl	0.020	0.020	0.018	0.017	0.018
	(0.021)	(0.021)	(0.023)	(0.021)	(0.021)
Number of older brothers (≤16y)				−0.030^**^	
				(0.014)	
Number of younger brothers (≤16y)				0.008	
				(0.013)	
Number of older sisters (≤16y)				−0.016	
				(0.018)	
Number of younger sisters (≤16y)				0.019	
				(0.013)	

*Village school quality*					
School quality index	0.012	0.013	0.009	0.014	0.036
	(0.014)	(0.014)	(0.014)	(0.014)	(0.081)
Observations	710	710	633	710	694
Village fixed effects included	No	No	No	No	Yes
Chi-square test for parental human capital marginal effect coefficients jointly = 0					
Chi-square statistic:	0.42	0.43	2.37		3.26
p > Chi-square:	0.811	0.809	0.668		0.196
Chi-square test for sibling marginal effect coefficients jointly = 0					
Chi-square statistic:				9.58	
p > Chi-square:				0.048	

Coefficients are average marginal effects of probit regressions. Standard errors clustered by household in parentheses. ^***^ p < .01, ^**^ p < .05, ^*^ p < .10. Definitions are provided in Table 3. In column 1, the omitted ethnicities are lowland, other ethnic group, and mixed (mother and father of different ethnicities).

## 6 Main regression results

The results are presented in Table 6, with variables grouped by hypotheses.

### 6.1 H1: Ethnic attributes

Our main test of the first hypothesis is reported in column 1 of Table 6. It shows support for the hypothesis that ethnicity is a predictor of participation in the CCT program for primary school attendance. Being a child in a household in which both parents are Tsimane’, the least-acculturated ethnic group, is associated with a large and statistically significantly lower probability of receiving a CCT payment, relative to all other ethnic groups. Children of Tsimane’ couples are 20 percentage points less likely to receive the educational cash transfer (p < .001). The size of coefficients varies in other regressions, but is always above 17 percentage points and statistically significant at the 5% level. On the other hand, children of Moxeño households, a group that has historically had more contact with mainstream Bolivian culture, are statistically equally likely to receive a CCT payment than lowlanders and “other” ethnic groups.

We probe further into the relation between CCT participation and ethnicity in two ways. First, we analyze the ethnicity of each parent separately (Appendix Table 4, columns 1 and 2). We find that the main result is driven by the ethnicity of the father. Having a Tsimane’ father is associated with a 17 percentage points lower probability of receiving an educational CCT payment (p = .002). The coefficient of the variable for Tsimane’ mothers is negative but not statistically significant at the 5% level (p = .083). Second, to assess if there are interaction effects between ethnicity and the other explanatory variables, we interact the variable indicating that parents are Tsimane’ with all the other explanatory variables (Appendix Table 4, column 3). The results show that there are interaction effects between a child’s age and having Tsimane’ parents, but there are no significant interaction effects between ethnicity and other characteristics, such as parents’ modern human capital, child sex, and village school quality. Because understanding the interaction of the Tsimane’ binary variable, age and age squared is not intuitive from the regression, we plotted the relation between ethnicity, age, and predicted likelihood of participating in the CCT program in Appendix Fig. 2. Appendix Fig. 2 shows that age and participation in the program are non-linearly associated for both Tsimane’ children and children of other ethnicities, and that participation rates are lower for Tsimane’ children at almost all ages.

### 6.2 H2. Parental modern human capital: Schooling and fluency speaking Spanish

#### 6.2.1 Schooling

Columns 1–3 of Table 6 contain the main results of the test of H2 on the relation between parental maximum schooling and children’s participation in the CCT program. Parental maximum schooling is defined as the maximum grade attainment for the two parents (Table 3). These results do not confirm our second hypothesis. The regression coefficients are small and not statistically significant, neither when considering parents’ maximum schooling grade (column 2) nor when accounting for each parent’s schooling separately (column 3). Controlling for the number of children in the household (column 4) or for village fixed effects also barely changes the coefficients from columns 1–2. Estimating the model with mothers’ modern human capital and fathers’ modern human capital also does not change the sign or the statistical significance of any coefficient (Appendix Table 5). These results show that parental schooling of mothers or fathers (conditioning on Spanish speaking language skills) does not affect the probability of receiving a CCT payment.

#### 6.2.2 Spanish

With one exception, we find that the maximum parental fluency in spoken Spanish is not an important predictors of a child participating in the CCT program. Maximum parental fluency is determined by the parent with the highest fluency level (Table 3). The coefficients of columns 1–2 and 4 suggest that having a parent who is fluent or nearly fluent in Spanish does not change the probability of a child participating in the CCT program. The results of the regression in column 5, which controls for village fixed effects, suggest that having a parent who is fluent or moderately fluent in Spanish may be associated with an increase in the likelihood that the child will participates in a CCT program, but the coefficient is only statistically significant at the 10% level (p = .079). The corresponding coefficients in other models are not statistically significant, calling for caution in giving too much weight to this result. Column 3 and Appendix Table 5 show that when we include variables for the fluency speaking Spanish of each parent separately, those results are still not statistically significant.

#### 6.2.3 Joint significance of both modern human capital variables

Chi-square tests of the joint significance of the variables for schooling and Spanish-speaking ability, in Table 6 and in Appendix Table 5, show that the two variables together were not significant predictors of CCT participation. In sum, we find no evidence that the level of modern human capital of parents predicts whether a child receives the CCT transfer.

### 6.3 H3. Child attributes.

Evidence for our third hypothesis is presented in Table 6, columns 1 and 4. As hypothesized, we do not find evidence of gender disparity in participation in the CCT programs. The point estimates suggest that girls are about 2 percentage points less likely to participate in the CCT program than boys, but results are not statistically significant (p = .338 and p = .411, columns 1 and 4).

Our hypothesis that participation increases with age is partially confirmed. Regression coefficients indicate that age is statistically significantly and non-linearly associated with participation in the CCT program. Dividing the negative of the coefficient for the linear term by twice the coefficient for the squared term suggests that participation increases with age up until the age of about 11 years, then decreases after that (averages in the raw data, shown in Appendix Fig. 1, place peak participation rate at 13 years).

We re-estimate the regression in column 1 with a series of variables indicating the number of older brothers, older sisters, younger brothers, and younger sisters of each child (Table 6, column 4). We do this because, as noted earlier, previous research among the Tsimane’ suggests that the sibling composition of the household predicts child educational attainment (Zeng et al., 2012). We find that the sibling composition of the household predicts participation, as evidenced by the statistically significant Chi-square test (χ^2^ = 9.58, p = .048). Specifically, the number of older brothers is a statistically significant predictor of participation in the CCT program, although the size of the relation is small, whereas the numbers of older sisters or younger siblings of both sexes are not. The coefficient estimate indicates that each additional older brother is associated with a 3 percentage points lower probability of receiving a CCT payment (p = .033), consistent with (Zeng et al., 2012). However, the average number of older brothers of children in our sample is only 0.5 (SD = 0.8), or 1.3 conditional on having an older brother, so in practice an average child with an older brother would in fact only be 3.9 percentage points less likely to participate in a CCT program.

### 6.4 Robustness test

To test the robustness of our findings, we use village fixed effects to control for village characteristics that may predict children’s participation in CCT programs and correlate with the covariates of interest (distinct from the schools’ characteristics included in the index of school quality). Some of these characteristics could include distance from villages to the nearby town, or village ethnic mix. Coefficient estimates from this regression are presented in Table 6, column 5. These results largely confirm our main findings. For example, after including village fixed effects, having Tsimane’ parents is associated with a 17.5 percentage points lower probability of receiving a CCT payment (p = .027). In this model, most parental and child characteristics also remain not statistically significant predictors of CCT participation, and age has the same nonlinear relation with participation. As in the previous regressions, school quality does not predict participation after controlling for other village characteristics.

## 7 Discussion of possible mechanisms for the findings

The Tsimane’ are the least acculturated of the ethnic groups in our sample; the significantly lower level of participation in the CCT program by Tsimane’ children, even after controlling for parental schooling and fluency in spoken Spanish and for school characteristics, suggests that the program does not fully redress inequalities in access to services that aim to be universal. We explore several mechanisms that could explain why Tsimane’ children have lower participation rates in the CCT program.

### 7.1 Program awareness

Raising awareness has been shown to increase participation in government programs in Latin America (Brandt, 2012, Larrañaga et al., 2012). Among the population we study, Reyes-García, Vadez, Aragon, Huanca, and Jagger (2010) show that the Tsimane’ have limited awareness of the Bolivian government’s decentralization reform, so one could argue that low awareness also explains Tsimane’ lower participation in the CCT program.

This interpretation has two main problems. First, awareness of the CCT program is likely to be higher than awareness of decentralization because CCT program pay families directly, and payment days are widely announced and result in salient festive occasions in villages (Zycherman, 2013). Second, villages in which Tsimane’ children live are smaller than villages in which children of other ethnic groups live. Living in smaller villages should make it easier for the school teacher to communicate the existence of the program and the schedule of payments within the village.

### 7.2 Limited parental modern human capital

Even if they are aware of the program, Tsimane’ parents, having limited ability to speak Spanish, could be hampered in their ability to interact with the school teacher(s), or with the military on payment days. In Mexico, de Janvry and Sadoulet (2006) found that indigenous parents’ literacy predicted their children’s participation in the CCT for primary school. However, our results show that this mechanism is likely not at play in Bolivia. The maximum school grade attainment or the ability to speak Spanish fluently of either parent or of both parents combined never predicted the participation of the child in the CCT program. Even if parents lack the modern human capital skills and comfort to communicate directly with teachers and the military, others in the village can inform unschooled parents of the program.

### 7.3 Within-village costs of reaching school

Since each village in our dataset has a school and payments are made in the village, the relevant transportation costs are within-village costs. Within a village, some children may face higher costs of reaching the school. Although villages are nucleated, not all households have the same physical access to the village school. Being less acculturated than other ethnic groups, Tsimane’ households may prefer living farther away from village centers, creating physical difficulties for their children to attend school and meet the program’s conditions. During most of the year, household-to-school distance is not a hurdle to school attendance, but during the rainy season when CCT payments happen, children who live far from school may find it harder to attend school because they cannot cross growing bodies of water. The smaller size of villages in which Tsimane’ children live (18 households/village on average, versus 36–54 households/village for children of other ethnicities; Table 5), however, again casts doubts on the effect of within-village transportation costs on school enrollment and CCT program participation. Our data do not include information on place of residence of each household within the village, house-to-school distance, travel time, or seasonal water barriers, so we cannot test this hypothesis empirically.

### 7.4 Culture and trust

Participation of Tsimane’ children could be affected by their own or their parents’ ability to interact with, cultural norms towards, or trust in government officials or outsiders to their communities. This mechanism is unlikely to interfere with the families’ ability to receive the payment, for two reasons: (i) the government officials most directly involved in administering the program and the payments at the village level are the school teachers, who are well known by and familiar to village inhabitants, and (ii) most Tsimane’ interactions with outsiders are non–hostile and welcoming, with the largest rejection corresponding to highland colonist farmers entering Tsimane’ territory (Reyes-García et al., 2012).

### 7.5 Utility of formal schooling

Because the Tsimane’ are more autarkic than the other groups, they might not derive as much utility from formal schooling. For example, Tsimane’ adults (>16 years) were 23% less likely than adults from other groups to earn any monetary income from wage labor or from the sale of farm goods. Because they are highly autarkic, they rely more on traditional forms of human capital for their livelihood and health, such as local ecological knowledge, and thus place less stress on formal schooling for their children (McDade et al., 2007, Reyes-García, 2015). Some evidence suggests that learning local ecological knowledge is not always compatible with investments in schooling (Reyes-García et al., 2007). The Tsimane’ also actively practice endogamy with respect to their ethnic group (Patel et al., 2007), which means that (i) Tsimane’ are less likely to connect with national society through marriage, and (ii) Tsimane’ society is tightly knit with very little market-oriented interactions within villages. For example, there are no shops in Tsimane’ villages, and locally-produced goods are more commonly bartered than sold (Undurraga, Zycherman, Yiu, & Godoy, 2014).

Lower returns to schooling do not imply that modern forms of human capital have no benefits for the Tsimane’. Studies have shown that schooling, fluency in Spanish, and math skills correlate positively with better market outcomes for the Tsimane’ (Godoy et al., 2007, Godoy et al., 2007; Undurraga et al., 2013). As we have documented above, however, modern human capital provides small benefits within villages or among the Tsimane’, so the returns to education are primarily limited to interactions outside the Tsimane’ culture or at least outside their villages. It is possible that the monetary cash transfer unlocked by children attending school and the limited benefits of schooling are not worth the opportunity cost of children attending school.

## 8 Conclusion

We estimate predictors of participation in a conditional cash transfer program in Bolivia which tries to increase child enrollment in primary school. Understanding participation matters because it can help improve the program and broaden its reach. Worryingly, our findings based on 811 school-aged children in rural areas in the Bolivian Amazon indicate that the most marginalized, least-acculturated, group (Tsimane’) is less likely to participate in an educational CCT program whose mission is, precisely, to increase enrollment. In the entire sample, children who did not participate in the program completed 30% fewer grades of schooling than children who participated, with a more pronounced effect for younger children. The most likely mechanism at play is that returns to formal schooling are lower for the Tsimane’ due to their autarkic, self-sufficient livelihoods.

Our findings raise the question of how to increase participation rates in CCT programs among ethnic groups least exposed to national society. We suggest three ways to increase participation. One is to increase the amount of the transfer, possibly combined with differentiated payments for different participants. The annual payment in Bolivia has been fixed at US$28 per child and has not changed since the program started. McGuire (2013) argues that the level is too low, as it fails to compensate households for the opportunity cost of their children going to school and other costs to participating. However, small transfers have been shown in other educational CCT programs to have large impacts (Kremer, Brannen, & Glennerster, 2013), which implies that even a small payment might incentivize participation. Higher payments may also not be feasible in limited national budgets. Differentiating payments by characteristics of the participants might be a workable solution to this problem (Dayioglu, Murat, & Aysit, 2009), although the evidence that differential payments increase participation is unclear (Baird et al., 2011, Filmer and Schady, 2011, Kremer et al., 2013). Finally, making school curricula more relevant for lowland indigenous children would increase the returns to schooling for these populations, could incentivize enrollment and attendance, and by consequence could increase participation in the program.

Another possible way to increase participation is to relax conditionality in the program. Relaxing conditionality can increase participation by reducing the burden of complying with conditions (Cookson, 2016, Ma et al., 2017). For example, relaxing conditionality may incentivize children to attend school at least some of the time. The traditional concern with removing conditionality is that it might decrease the effectiveness of the program, but evidence from randomized controlled trials and meta-analyses suggest that the impacts of unconditional cash transfer programs in education and in other sectors are positive (Benhassine et al., 2015, Haushofer and Shapiro, 2016), and not statistically significantly lower than those of conditional cash transfer programs (Baird et al., 2014, Manley et al., 2013).

Conditional cash transfer programs have generally proven effective at increasing primary-school enrollment and attendance among low-income groups. These programs may have long-lasting effects on children and their households. But our findings indicate that reaching the most marginalized, disadvantaged ethnic minorities may require additional efforts at reducing barriers to participation.
