# Supplementary material for: Conditional cash transfers for primary education: Which children are left out?
Source: World Dev. 2018 May;105:1–12. doi: 10.1016/j.worlddev.2017.12.021 (PMC6472288; doi:10.1016/j.worlddev.2017.12.021)
Supplement: Supplementary data 1 [file mmc1.pdf]

Conditional cash transfers for primary education: Which children are left out?

Supplemental File

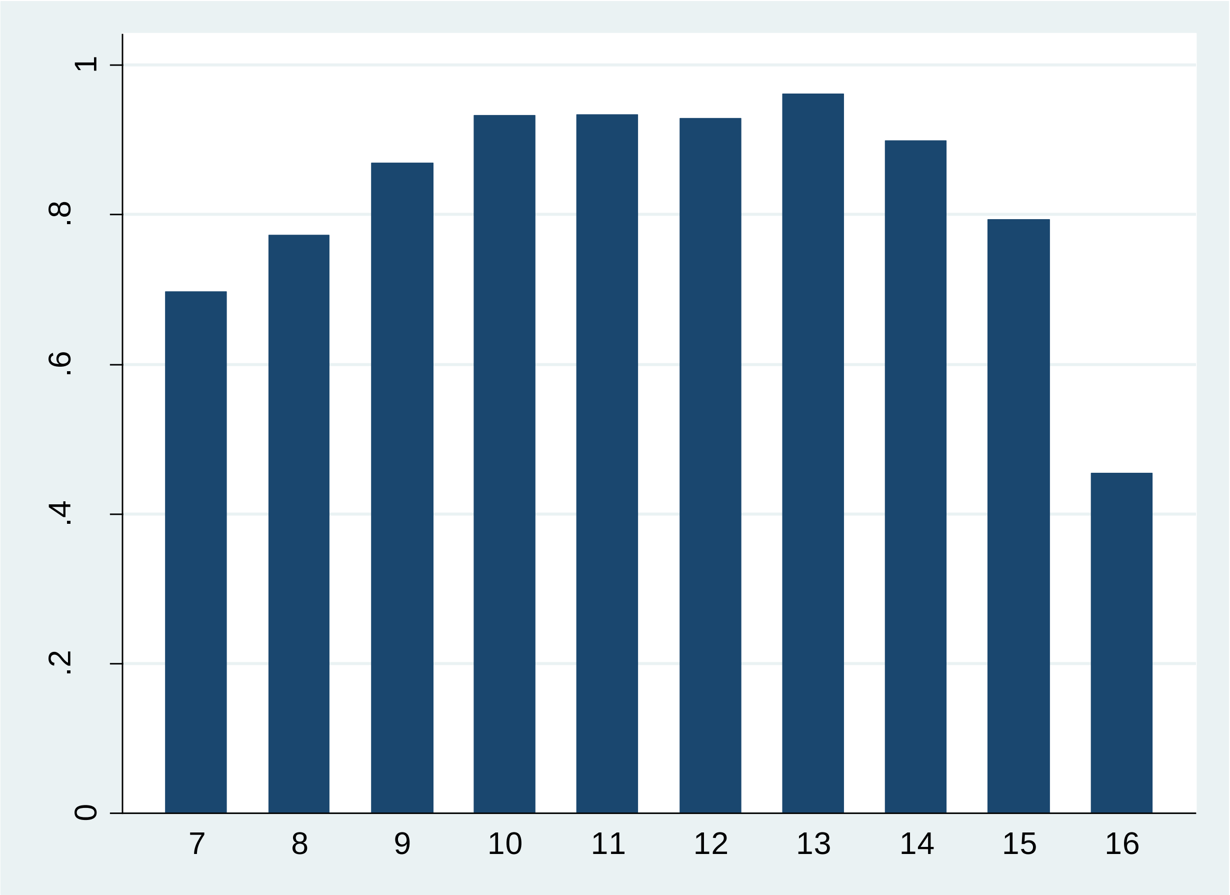

Appendix Figure 1. Participation in CCT program, by child age (in years).

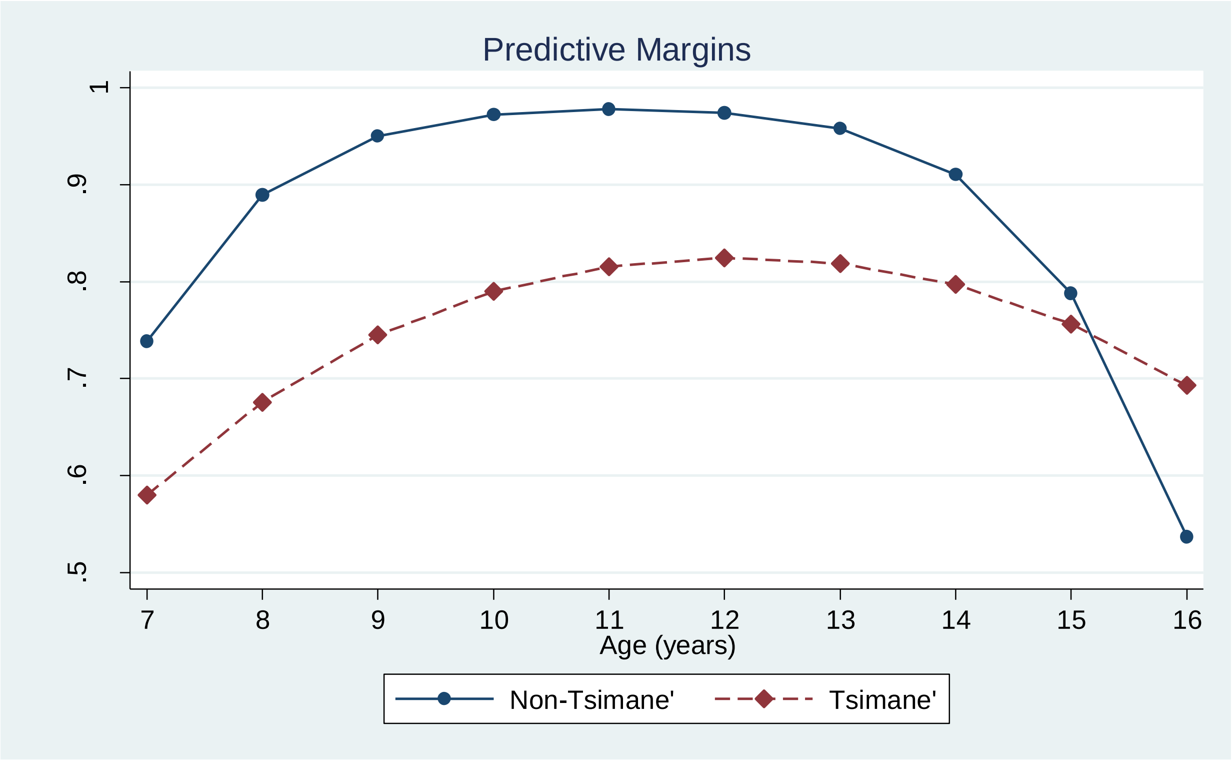

Appendix Figure 2. Predicted rates of participation in the CCT program, by age and ethnicity.

Appendix Table 1. Correlation coefficients between main variables in the regression analyses.

	Child received payment from CCT program	Parents are both Moxeño	Parents are both Tsimane'	Max. schooling of parents	Max. Spanish fluency of parents	Age	Girl	School quality index
Child received payment from CCT program	1							
Parents are both Moxeño	0.1799***	1						
Parents are both Tsimane'	-0.2703***	-0.5919***	1					
Max. schooling of parents	0.1356***	0.2759***	-0.4252***	1				
Max. Spanish fluency of parents	0.218***	0.4642***	-0.7802***	0.4366***	1			
Age	0.0521	0.1105*	-0.0911	-0.0449	0.0568	1		
Girl	0.0428	-0.0094	-0.0125	-0.0141	0.0215	0.0463	1	
School quality index	0.1549***	0.3556***	-0.4571***	0.3812***	0.4117***	0.0461	0.0228	1

*** p<0.01, ** p<0.05, * p<0.10.

Appendix Table 2. Variance inflation factors after regressions displayed in Table 6.

	(1a)	(1b)	(2a)	(2b)	(3a)	(3b)	(4a)	(4b)	(5a)	(5b)
Dependent variable:	1 if child received payment from CCT program; 0 otherwise									
**H1: Ethnic attributes - Household ethnicity**										
Parents are both Tsimane’	3.16	3.16	2.67	2.67	2.58	2.58	2.72	2.72	7.68	7.68
Parents are both Moxeño	1.57	1.57								
**H2: Parental modern human capital**										
Max. schooling of parents	1.34	1.33	1.34	1.33			1.39	1.39	1.40	1.40
Max. Spanish fluency of parents	2.68	2.68	2.68	2.68			2.76	2.76	3.50	3.50
Mother: Schooling					2.06	2.06				
Mother: Speaks Spanish					4.16	4.14				
Father: Schooling					1.92	1.92				
Father: Speaks Spanish					3.94	3.92				
**H3: Child attributes**										
Age (years)	80.81	1.03	80.81	1.02	79.80	1.05	81.18	1.61	80.81	1.07
Age squared	80.84		80.84		79.87		81.51		80.77	
Girl	1.01	1.00	1.01	1.00	1.01	1.01	1.01	1.01	1.05	1.05
Number of older brothers ≤16y							1.19	1.19		
Number of younger brothers ≤16y							1.11	1.11		
Number of older sisters ≤16y							1.30	1.30		
Number of younger sisters ≤16y							1.10	1.10		
**Village school quality**										
School quality index	1.28	1.28	1.27	1.26	1.30	1.30	1.29	1.29	73.13	73.05
Observations	710		710		633		710		694	
Village fixed effects included	No		No		No		No		Yes	
Maximum VIF on village dummies	-		-		-		-		41.29	41.21

Columns go by pair, showing the VIFs after regressions in columns (1) to (6) of Table 5. “a”-numbered columns in this table show the VIFs including age square in the regression; “b”-numbered columns in this table show the VIFs without age square in the regression. The next largest VIFs of village dummy variables are 3.04 in column (5a) and 3.03 in column (5b).

Appendix Table 3. Correlates of child CCT participation, various configurations of parental education variables.

	(1)	(2)	(3)	(4)
Specification:	Main model (Table 6, col. 1)	No parental modern human capital	Parental schooling only	Parental Spanish only
Dependent variable:	1 if child received payment from CCT program; 0 otherwise			
**H1: Ethnic attributes - Household ethnicity**				
Parents are both Tsimane’	-0.200***	-0.175***	-0.181***	-0.198***
	(0.056)	(0.045)	(0.048)	(0.056)
Parents are both Moxeño	0.020	0.020	0.020	0.020
	(0.035)	(0.035)	(0.035)	(0.035)
**H2: Parental modern human capital**				
Max. schooling of parents	-0.001		-0.002	
	(0.005)		(0.004)	
Max. Spanish fluency of parents	-0.026			-0.029
	(0.053)			(0.051)
**H3: Child attributes**				
Age (years)	0.279***	0.280***	0.279***	0.280***
	(0.035)	(0.035)	(0.035)	(0.035)
Age, squared	-0.012***	-0.012***	-0.012***	-0.012***
	(0.002)	(0.002)	(0.002)	(0.002)
Girl	0.020	0.021	0.020	0.021
	(0.021)	(0.021)	(0.021)	(0.021)
**Village school quality**				
School quality index	0.012	0.011	0.012	0.012
	(0.014)	(0.014)	(0.014)	(0.014)
Observations	710	710	710	710
Village fixed effects included	No	No	No	No

Coefficients are average marginal effects of probit regressions. Standard errors clustered by household in parentheses. *** p<0.01, ** p<0.05, * p<0.10. Definitions are provided in Table 3. In column 1, the omitted ethnicities are lowland, other ethnic group, and mixed (mother and father of different ethnicities).

Appendix Table 4. Correlates of child CCT participation, mother's and father's ethnicity and interaction of ethnicity with other variables.

	(1)	(2)	(3)
Dependent variable:	1 if child received payment from CCT program; 0 otherwise		
**H1: Ethnic attributes - Household ethnicity**			
Mother is Tsimane'	-0.121*		
	(0.0697)		
Mother is Moxeño	0.0153		
	(0.0501)		
Father is Tsimane'		-0.171***	
		(0.0547)	
Father is Moxeño		0.0242	
		(0.0392)	
Parents are both Tsimane'			4.169
			(2.853)
**H2: Parental modern human capital**			
Max. schooling of parents	0.00253	-0.00157	-0.008
	(0.00468)	(0.00446)	(0.0268)
Max. Spanish fluency of parents	0.0601	-0.00602	-0.356
	(0.0525)	(0.0478)	(0.513)
Max. schooling of parents * Tsimane'			-0.0432
			(0.104)
Max. Spanish fluency of parents * Tsimane'			0.243
			(0.629)
**H3. Child attributes**			
Age (years)	0.284***	0.291***	1.728***
	(0.0345)	(0.0343)	(0.236)
Age squared	-0.0126***	-0.0128***	-0.077***
	(0.00153)	(0.00153)	(0.0104)
Girl	0.0264	0.0217	0.175
	(0.0206)	(0.0208)	(0.136)
Age (years) * Tsimane'			-1.004*
			(0.524)
Age squared * Tsimane'			0.0473**
			(0.0231)
Girl * Tsimane'			-0.261
			(0.291)
**Village school quality**			
School quality index	0.0106	0.0116	0.0646
	(0.0133)	(0.0134)	(0.0784)
School quality index * Tsimane'			0.562
			(0.641)
Observations	781	737	710
Village fixed effects included	No	No	No

Coefficients in columns (1) and (2) are average marginal effects of probit regressions; coefficients in column (3) are probit coefficients. Standard errors clustered by household in parentheses. *** p<0.01, ** p<0.05, * p<0.10. Definitions are provided in Table 3. The omitted ethnicities in columns (1) and (2) are lowland, other ethnic group, and mixed-ethnicity couples; in column (3) Moxeño is also omitted (for clarity and ease of interpretation of the interaction terms, since being of Moxeño ethnicity is not related to participation in the CCT program).

Appendix Table 5. Correlates of child CCT participation, mothers' and fathers' modern human capital.

	(1)	(2)
Dependent variable:	1 if child received payment from CCT program; 0 otherwise	
**H1: Ethnic attributes - Household ethnicity**		
Parents are both Tsimane'	-0.178***	-0.172**
	(0.055)	(0.068)
**H2: Parental modern human capital**		
Mother: Schooling	-0.004	
	(0.005)	
Mother: Speaks Spanish	0.034	
	(0.050)	
Father: Schooling		0.001
		(0.005)
Father: Speaks Spanish		0.027
		(0.066)
**H3: Child attributes**		
Age (years)	0.276***	0.285***
	(0.036)	(0.037)
Age squared	-0.012***	-0.013***
	(0.002)	(0.002)
Girl	0.016	0.023
	(0.021)	(0.023)
**Village school quality**		
School quality index	0.012	0.007
	(0.014)	(0.014)
Observations	710	710
Village fixed effects included	No	No
Chi-square test for parental human capital marginal effect coefficients jointly=0		
Chi-square statistic:	0.84	0.20
p>Chi-square:	0.658	0.903

Coefficients are average marginal effects of probit regressions. Standard errors clustered by household in parentheses. *** p<0.01, ** p<0.05, * p<0.10. Definitions are provided in Table 3. The omitted ethnicities are Moxeño, lowland, other ethnic group, and mixed-ethnicity couples.

Appendix Table 6. Correlates of child CCT participation, including travel time to nearest town.

	(1)	(2)
Dependent variable:	1 if child received payment from CCT program; 0 otherwise	
**H1: Ethnic attributes - Household ethnicity**		
Parents are both Tsimane’	-0.211***	-1.094
	(0.061)	(1.043)
Parents are both Moxeño	0.007	0.414
	(0.043)	(0.437)
Parents are both Tsimane’ * Time to town		-0.010
		(0.072)
Parents are both Moxeño * Time to town		-0.038
		(0.043)
**H2: Parental modern human capital**		
Max. schooling of parents	-0.002	-0.021
	(0.006)	(0.063)
Max. Spanish fluency of parents	-0.053	-0.323
	(0.052)	(0.834)
Max. schooling of parents * Time to town		0.002
		(0.005)
Max. Spanish fluency of parents * Time to town		-0.001
		(0.039)
**H3. Child attributes**		
Age (years)	0.283***	1.891***
	(0.042)	(0.454)
Age squared	-0.013***	-0.082***
	(0.002)	(0.020)
Girl	0.007	-0.340
	(0.025)	(0.237)
Age (years) * Time to town		-0.030
		(0.029)
Age squared * Time to town		0.001
		(0.001)
Girl * Time to town		0.024*
		(0.014)
**Village school quality and Travel time to nearest town**		
School quality index	0.014	-0.438
	(0.015)	(0.314)
School quality index * Time to town		0.042*
		(0.024)
Travel time to nearest town, walking, dry season (hours)	-0.003	0.190
	(0.002)	(0.185)
Observations	537	537
Village fixed effects included	No	No

Coefficients in column (1) are average marginal effects of probit regressions; coefficients in column (2) are probit coefficients. Standard errors clustered by household in parentheses. *** p<0.01, ** p<0.05, * p<0.10. Definitions are provided in Table 3. The omitted ethnicities are lowland, other ethnic groups, and mixed (mother and father of different ethnicities).
